# Broiler chicken carcasses and their associated abattoirs as a source of enterotoxigenic *Clostridium perfringens*: Prevalence and critical steps for contamination

**DOI:** 10.3934/microbiol.2018.3.439

**Published:** 2018-06-13

**Authors:** Marie-Lou Gaucher, Alexandre Thibodeau, Philippe Fravalo, Marie Archambault, Julie Arsenault, Sylvain Fournaise, Ann Letellier, Sylvain Quessy

**Affiliations:** 1 Research Chair in Meat Safety, Département de Pathologie et Microbiologie, Faculté de médecine vétérinaire, Université de Montréal, 3200 Sicotte, St-Hyacinthe, Québec, Canada; 2 Swine and Poultry Infectious Diseases Research Centre (CRIPA), Faculté de médecine vétérinaire, Université de Montréal, 3200 Sicotte, St-Hyacinthe, Québec, Canada; 3 Olymel S.E.C./L.P., Québec, Canada, 2200 Avenue Léon-Pratte, St-Hyacinthe, Québec, Canada

**Keywords:** broilers, slaughterhouse, steps, *Clostridium perfringens*, enterotoxin, prevalence

## Abstract

*Clostridium perfringens* ranks among the three most frequent bacterial pathogens causing human foodborne diseases in Canada, and poultry meat products are identified as a source of infection for humans. The objective of the current study was to estimate the proportion of broiler chicken flocks, carcasses and various environmental samples from critical locations of the slaughter plant positive for the presence of *C. perfringens* enterotoxin encoding gene (*cpe*). From the 16 visits conducted, 25% of the 79 flocks sampled, 10% of the 379 carcasses sampled and 5% of the 217 environmental samples collected were found positive for *cpe.* The proportion of *cpe*-positive carcasses was statistically different between surveyed plants, with 17.0% for one abattoir and 2.2% for the other. For the most contaminated plant, *cpe*-positive carcasses were identified at each step of the processing line, with prevalence varying between 10.0% and 25.0%, whereas this prevalence varied between 0% and 25.0% for the environmental surfaces sampled. Based on the results obtained, enterotoxigenic *C. perfringens* strains could potentially represent a risk for the consumer.

## 1. Introduction

It is estimated that approximately 4 million Canadians suffer from food poisoning each year, and *Clostridium perfringens* has been identified as one of the bacterial pathogens causing the greatest number of these illnesses [1]. Indeed, based on Canadian data, *C. perfringens* ranks second as the cause of domestically acquired food poisoning illnesses, with an estimated number of 544.5 illness cases/100,000 inhabitants [2]. Similarly, in the United States, *C. perfringens* is responsible for nearly 1 million disease cases per year [3]. As with most of other toxin-producing bacterial pathogens, the main transmission pathway of *C. perfringens* is food, with meat representing an important vehicle [4]. Ribotyping analysis revealed poultry meat as a source of *C. perfringens* in human foodborne disease outbreaks [5]–[14], but few studies have investigated the presence of *cpe*-carrying *C. perfringens* in processed poultry meat products. While recent studies have shed some light on the ecology of CPE-producing *C. perfringens* strains, much work remains to be done to better describe the epidemiology of this pathogen, from its reservoirs to the human digestive tract [15]–[18]. *C. perfringens* is widely distributed in soils, wastewaters, foods, in addition to being part of the normal intestinal microflora of animals and humans [19]. Classically, strains of this bacterial species are categorized into five types, from A to E, based on the carriage of different combinations of the four toxin-encoding genes named *cpa*, *cpb*, *etx* and *iA* [20]. At least 17 different toxins have been described in *C. perfringens* so far, and among those, the enterotoxin (CPE) encoded by the *cpe* gene is accountable for the intestinal symptoms associated with *C. perfringens* food poisoning in humans [8],[20]. Following ingestion of *C. perfringens* vegetative cells, sporulation-initiating factors present in the human intestinal environment will induce the simultaneous sporulation and CPE production by the bacterium. Upon lysis, these cells will release large amounts of CPE molecules into the intestinal lumen and the typical symptoms of a gastro-intestinal illness will appear following the binding of these CPE molecules to specific intestinal epithelial receptors [16],[20],[21].

Steps of the slaughter process that are critically impacting broiler chicken carcasses contamination by CPE-producing *C. perfringens* fully remain to be identified. As is the case for other pathogens, enterotoxigenic *C. perfringens* could contaminate poultry meat either because of intestinal content spillage following evisceration, or through contact with contaminated work surfaces [13],[22]–[24]. As opposed to *Salmonella* spp. and *Campylobacter* spp. for which studies have clearly identified the sources of contamination and the critical points to be controlled and monitored at the various stages of the slaughter process [25]–[30], no study has investigated broiler chicken carcasses as a potential source of enterotoxigenic *C. perfringens*.

Modern poultry slaughtering rely on highly automated processes and equipment, and preventing bacterial contamination of poultry carcasses is a constant challenge for processors who must comply with regulatory guidelines [31]. In this regard, the identification of critical steps of the slaughter process that are impacting the contamination of poultry carcasses by enterotoxigenic *C. perfringens* will help implement control measures at the slaughterhouse level, ultimately contributing to decrease poultry meat contamination by this zoonotic pathogen.

Most of the studies conducted so far have looked at the presence of enterotoxigenic *C. perfringens* strains in poultry meat products using a classical culture approach, including the selection of suspect colonies based on their haemolytic activity on blood agar [5]–[13]. However, as less than 5% of all type A *C. perfringens* strains are reported to carry *cpe*, and as traditional culture methods do not allow for the distinction between *cpe*-positive and *cpe*-negative isolates, a stringent approach is required to detect and isolate these strains from any analyzed samples [15],[16]. Indeed, the double hemolysis typically produced by *C. perfringens* on blood agar during standard isolation procedures and attributable to the theta toxin gene (*pfoA*) can't be used as a criterion for selecting typical *cpe*-positive *C.* *perfringens* colonies during final steps of the isolation protocol. The absence of *pfoA* in a large proportion of *cpe*-positive *C. perfringens* strains is thus contributing to distort the study of the epidemiology of this foodborne pathogen [16],[32]. Accordingly, establishing with certainty the role of poultry in the dissemination of *cpe*-carrying *C. perfringens* is of great challenge. By using a different approach, the aims of the current study were then, to estimate and compare the proportion of broiler chicken carcasses positive for the presence of the enterotoxin-encoding gene, *cpe*, at critical steps of the slaughter process, and to investigate the presence of this pathogen in the environment of two poultry slaughter plants in Québec, Canada.

## 2. Materials and methods

### 2.1. Processing plant and flock selection

Two different poultry processing plants in the province of Québec, Canada, were sampled between the months of February and July of 2017. Each surveyed plant's operation and sanitation program characteristics are presented in Table 1. For both slaughter plants, sampling visits were scheduled according to the number of flocks slaughtered per day, with a minimum number of five different commercial broiler chicken flocks that needed to be available for a sampling visit to be conducted in a surveyed plant. During each visit, five different flocks were sampled.

### 2.2. Sampling procedures

#### 2.2.1. Whole carcass sampling

Whole carcasses were randomly chosen from the last one-third of each sampled flock. For each flock, one carcass was sampled from five different critical points of the processing line: after bleeding, with feathers still attached to the carcass (B), before evisceration (BE), before chilling (BC), after immersion water (W) and dry-air (A) chilling [29]. A total of 5 birds were sampled for a same flock; however, only four birds were sampled in flocks from which air-chilled carcasses were not available. The methodology suggested by the U.S. Department of Agriculture Food Safety and Inspection Service for collecting raw meat and poultry product samples was applied, with slight modifications with regard to the volume of buffered peptone water used [33]. Each sampled carcass was placed in a sterile bag (Fisher Scientific, Ottawa, Nasco Poultry Rinse Sample Bag) and rinsed through vigorous shaking with 550 mL of buffered peptone water (Lab M Ltd., Heywood, UK) for 1 min. Collected rinsates were placed on ice and transported back to the laboratory where further analysis was performed.

**Table 1.** Surveyed abattoirs' operation and sanitation program management characteristics.

Operation characteristics	Abattoirs	
	Plant A	Plant B
Slaughtering description		
No. of live haul transportation companies	3	4
No. of production types slaughtered	2	4
Average number of flocks slaughtered/day	10	9
Average number of birds/flock	25,000	25,000
Male/Female ratio for slaughtered birds	2.2:1.0	1.0:3.0
Processing description		
Line speed	225 birds/min	230 birds/min
No. of scalding tanks	3	2
Minimum scalding temperature	53.3 °C	50.0 °C
Maximum scalding temperature	57.2 °C	61.7 °C
Scalding time	1 min 30 sec	1 min 20 sec
Plucking time	35 sec	26 sec
No. carcass washes along the processing line	6	Between 4 (immersion) and 9 (air)
Carcass sanitizer	Quaternary ammonium	Peracetic acid
Type of immersion water chiller	Not counter-flow	Counter-flow
Immersion water chiller tank temperature	1 °C–3 °C	1 °C
Time in immersion water chiller	1 h 30 min	1 h 50 min
Air temperature in dry-air chilling room	−3 °C–2 °C	0.6 °C
Time in dry-air chilling room	1 h 30 min	1 h 47 min
Plant sanitation		
Sanitizer	Organic, inorganic acids, hydrogen peroxyde	Quaternary ammonium

#### 2.2.2. Environmental sampling

Both processing plants were also investigated through environmental samplings that were conducted after the sanitation procedures, prior to the slaughter activities (PS), as well as at the end of the work shift (OP). Those samplings were conducted during each visit, on seven critical sampling locations (CSLs). According to Luning et al. [34], CSLs were defined as surfaces on which contamination, growth and survival of microorganisms can occur due to the type of operations performed, or to the risk of the applied prevention strategies not being fully effective in controlling contamination. Those CSLs included: the feather-plucking rubber fingers (E1), the conveyor belt between the live receiving and evisceration departments (E2), the evisceration machine (E3), the floor surface in the evisceration department (E4), the conveyor belt before chilling (E5), the conveyor belt after chilling (E6) and a stainless steel equipment surface located in the cut-up room (E7). Briefly, individual sterile 8.5 × 9.5 in gauze pads moistened in a 10 mL neutralizing buffer volume (DE Neutralising Broth, LabM, Ltd. UK) were used to vigorously rub a 10 cm × 10 cm surface on each CSL. After sampling, gauze pads were put back into their respective sterile bags, placed on ice and transported to the laboratory for microbiological analysis.

### 2.3. Sample treatment and microbiological procedures

#### 2.3.1. Broiler carcass rinsates

Upon arrival at the laboratory, all samples were stored overnight, at 4 °C, and were individually processed the morning after. From the initial 550 mL rinsate volume, 200 mL were centrifuged (ThermoFisher Sorvall Legend XTR) for 20 min at 15,000 g (ThermoFisher Fiberlite F14-6 × 250 LE Rotor). Following centrifugation, the supernatant was aseptically removed and the pellet resuspended in a 4 mL volume of buffered peptone water (Lab M Ltd.). All samples were vortexed until complete dissolution of the pellet. One mL of this suspension was distributed in 9 mL of fluid thioglycolate medium (FTG) with resazurin (Biokar Diagnostics, Cedex, France) and was homogenized. Each sample was duplicated by transferring 5 mL of the original 10 mL volume into a sterile tube. For each sample, one replicate was submitted to a heat treatment, at 72 °C for 20 min, in order to allow the germination of spores that could have been present in the sample. Both heat-treated and non-heat-treated tubes were incubated at 37 °C, for 24 h, under anaerobic conditions (AnaeroGen Gas Generating System, Oxoid, Ontario, Canada).

#### 2.3.2. Environmental samples

Similarly, environmental samples collected during each sampling visit were kept refrigerated and were processed in the laboratory on the subsequent day. A 9 mL volume of FTG with resazurin (Biokar Diagnostics) was added to each gauze-containing bag before stomaching for one minute. Each bag was then squeezed in one hand to allow the recovery of the whole 9 mL initial volume of FTG medium that was immediately distributed into sterile tubes. Again, each sample was duplicated into two 4.5 mL volumes, and one replicate was submitted to the same heat treatment at 72 °C for 20 min, for the reason mentioned in 2.3.1. Both heat-treated and non-heat-treated tubes were incubated at 37 °C, for 24 h, under anaerobic conditions (AnaeroGen Gas Generating System, Oxoid, Ontario, Canada).

### 2.4. DNA extraction and detection of cpe using a PCR-based approach

The same PCR-based detection approach for *cpe* was applied to all incubated samples. Briefly, for a same sample, FTG tubes were vortexed and 1 mL of each of the heat-treated and non-heat-treated tubes were pooled in a 2 mL microcentrifuge tube. The InstaGene matrix DNA extraction protocol using a 10% Chelex 100 solution in water was applied to these samples according to the manufacturer's instructions (Bio-Rad, Mississauga, Ontario) [6]. Briefly, 1 mL of each of the heat-treated and non-heat-treated tubes of a same sample were pooled in a 2 mL microcentrifuge tube and pelleted by centrifugation at 12,000 g for 1 min. The supernatant was removed and the pellet was washed once with a 1 mL volume of sterile water. After a second centrifugation, the supernatant was removed before adding 200 µL of 10% Chelex 100 solution. Microcentrifuge tubes were vortexed and incubated at 56 °C for 30 min. After incubation, tubes were placed in boiling water (100 °C) for 8 min. After boiling, microcentrifuge tubes were vortexed and their content was centrifuged at 12,000 g for 3 min. A hundred µL volume of the resulting supernatant was transferred into a sterile microcentrifuge tube and was used as DNA template. Primers used and PCR conditions were conducted according to the protocol published by Guran and Okzustepe [5], with slight modifications to allow smaller sample and reaction volumes. Each PCR reaction was conducted in a 25 µL reaction volume made of 15 µL of sterile water, 1× of reaction buffer (10× ThermoPol Reaction Buffer, NEB, Canada), 0.2 µM of dNTPs (Bio Basic Inc. Ontario, Canada), 2.5 U of Taq DNA Polymerase (NEB, Canada), 1 µL of each primer at 10 µM and 5 µL of template DNA. DNA amplification reactions were carried out using a Roche LightCycler® 96 Real-Time PCR thermocycler (Roche Diagnostics, Laval, Canada) and reaction conditions were as follows: an initial denaturation step at 94 °C for 2 min followed by 35 cycles of: denaturation at 94 °C for 1 min, annealing at 55 °C for 1 min, extension at 72 °C for 1 min. A final extension step at 72 °C for 10 min was also performed. Ten microliters of the PCR amplified products were visualized under UV light following electrophoresis on a 1% agarose gel containing 0.01% SYBR Safe DNA gel stain (Invitrogen, Burlington, Canada). A 100 bp ladder (Track It, Invitrogen, Burlington, Canada) was used as a molecular weight marker. Extracted DNA from *C. perfringens* type E (AHL#155, positive for *cpa*, *iA*, *cpe* and *cpb2* genes) and *C. perfringens* type A (AHL311, positive for *cpa* and *cpe* genes) was used as positive controls [35].

### 2.5. PCR product purification, sequencing, sequence alignment and comparison

The nature of the amplified PCR products visualized on agarose gel was confirmed through sequencing. PCR products were purified using the QIAquick® PCR Purification Kit and this was done according to the manufacturer's instructions (Qiagen, Canada). Purified PCR products were sequenced from both ends using the primers previously described (*cpe*F-GGAGATGGTTGGATATTAGG and *cpe*R-GGACCAGCAGTTGTAGATA) [5]. Between 3–20 ng template DNA and the ABI PrismÒ BigDye® Terminator Cycle Sequencing Ready Reaction kit v3.1 (Applied Biosystems, Foster City, CA) were used for sequencing reactions. The sequencing cycle was performed on a GeneAmp® PCR System 9700 or 2720 Thermal Cycler (Applied Biosystems). The BigDye® Terminator v3.1 Cycle Sequencing Kit Protocol, Rev A (Applied Biosystems) was followed to set up and conduct the cycle sequencing reactions. Dye terminators were removed from the cycle sequencing reactions using Multiscreen-HV plates (Millipore, Mississauga, ON) loaded with Sephadex G-50 superfine (Sigma, Oakville, ON). The clean reactions were electrophoresed on an Applied Biosystems 3730 DNA Analyzer (Applied Biosystems). A minimum read length of 700 bp was generated for each of the reactions. The chromatograms were analyzed using ABI PrismÒ DNA Sequencing Analysis Software Version 4 (Applied Biosystems) to generate quality target sequences within the Software's clear confidence range. To confirm the identity of the sequenced products, resulting sequences were aligned to the *cpe* gene sequence of the type A food poisoning reference strain SM101 of *C. perfringens* (https://www.ncbi.nlm.nih.gov/nuccore/CP000312.1) on the BLAST program (http://www.ncbi.nlm.nih.gov/). This same program was also used to align the sequences obtained with the information available on NCBI (http://www.ncbi.nlm.nih.gov/).

### 2.6. Statistical analysis

Descriptive statistics were used to present the results. A multivariable logistic regression was used to model *cpe*-positivity of carcasses according to the slaughter plant (A v. B) and to critical steps (B, BE, BC, W and A), with standard errors adjusted for clustering within flocks. A second multivariable logistic regression was used to model *cpe*-positivity of flocks, defined as the presence of at least one *cpe-*positive carcass, according to the sequential order of slaughtering (categorized from 1 to 5) and slaughter plant (A v. B), with standard errors adjusted for clustering within visit. A multivariable exact logistic regression was used to model *cpe*-positivity of environmental samples according to the slaughter plant (A v. B), the surface (7 categories) and the sampling period (post-sanitation v. operations). No adjustment was made for clustering within the sampling visit due to insufficient sample size, and because preliminary analyses showed an absence of association between the sampling day and *cpe*-positivity in environmental samples based on an exact chi-square test. An alpha value of 0.05 was used to determine statistical significance. All statistical analyses were conducted in SAS 9.4.

## 3. Results

### 3.1. Whole broiler chicken carcasses and environmental samplings

A total of 79 flocks, 379 broiler chicken carcasses and 217 environmental surfaces were sampled over a six-month period in the two surveyed slaughter plants (see Table 2). Both abattoirs were visited 8 times each (see Table 2). A total of 40 carcass rinsates were recovered from each of the five critical steps in abattoir A. For the carcass sampling conducted in abattoir B, bleeding (B), before evisceration (BE) and before chilling (BC) critical steps of the slaughter process were uniformly sampled during the study, with a total of 39 samples each, while 38 and 24 samples were recovered from the water immersion (W) and dry-air (A) chilling critical steps, respectively. A total number of 112 environmental samples were collected from both plants (56 from each) at the end of the slaughter activities, with 8 samples corresponding to each of the CSLs examined. The sampling conducted prior to the start of the slaughter operations included 8 samples per CSL in abattoir B, and 7 samples per CSL in abattoir A for which one post-sanitation sampling could not be conducted due to an earlier start of the operations on one visit day.

### 3.2. Detection of cpe-positive carcasses along critical steps of the processing line

From the 379 carcasses sampled, 38 (10.0%) were found to be positive for the presence of the *cpe* gene following enrichment culture and PCR steps. A higher odd of *cpe*-positivity was found in carcasses slaughtered in abattoir A (odds ratio = 8.8, p < 0.001). Indeed, 17.0% of carcasses were *cpe*-positive for slaughter plant A compared to 2.2% in abattoir B (see Table 1). No statistically significant difference in *cpe*-positivity of carcasses was found between sampled critical steps of the processing line (p = 0.23, 4 d.f.). *cpe*-positive broiler chicken carcasses were identified for every sampled critical step of the processing line and 15% of the carcasses sampled after chilling were found to be *cpe* positive.

**Table 2.** Numbers of flocks, carcasses and environmental samples studied and percentages of *cpe*-positive samples per slaughter plant and sampling critical points and locations.

	Abattoir A			Abattoir B			All	
	No. samples	% *cpe*-positive		No. samples	% *cpe*-positive		No. samples	% *cpe*-positive
Carcass sampling at critical point of the processing line								
After bleeding (B)	40	10.0		39	0.0		79	5.1
Before evisceration (BE)	40	17.5		39	2.6		79	10.1
Before chilling (BC)	40	10.0		39	0.0		79	5.1
After immersion water chilling (W)	40	22.5		38	5.3		78	14.1
After dry-air chilling (A)	40	25.0		24	4.2		64	17.2
*All*	*200*	*17.0*		*179*	*2.2*		*379*	*10.0*
Environmental sampling—Post-sanitation								
Feather-plucking rubber fingers (E1)	7	0		8	0		15	0
Conveyor belt: live receiving to evisceration (E2)	7	0		8	0		15	0
Evisceration machine (E3)	7	14.3		8	0		15	6.7
Floor surface evisceration (E4)	7	14.3		8	0		15	6.7
Conveyor belt before chilling (E5)	7	0		8	0		15	0
Conveyor belt after chilling (E6)	7	0		8	12.5		15	6.7
Stainless steel cut-up room (E7)	7	14.3		8	12.5		15	13.3
*All*	*49*	*6.1*		*56*	*3. 6*		*105*	*4. 8*
Environmental sampling—Operations								
Feather-plucking rubber fingers (E1)	8	0		8	0		16	0
Conveyor belt: live receiving to evisceration (E2)	8	12.5		8	0		16	6.3
Evisceration machine (E3)	8	12.5		8	0		16	6.3
Floor surface evisceration (E4)	8	0		8	0		16	0
Conveyor belt before chilling (E5)	8	0		8	0		16	0
Conveyor belt after chilling (E6)	8	12.5		8	12.5		16	12.5
Stainless steel cut-up room (E7)	8	25.0		8	0		16	12.5
*All*	*56*	*8.9*		*56*	*1. 8*		*112*	*5.4*

Even though no statistically significant difference was found between sampled critical steps of the slaughter process regarding the number of *cpe*-positive carcasses identified for both abattoirs, it is however interesting to mention that fewer *cpe*-positive broiler chicken carcasses were identified at bleeding (B) and before chilling (BC) critical steps of the processing line. Indeed, a total of eight carcasses out of the 34 identified as *cpe*-positive were found for both of these two critical steps of the process in plant A, whereas none was recovered from plant B. Table 3 shows the sequential order of the detection of *cpe*-positive broiler chicken carcasses and CSL samples among sampling dates, critical steps of the processing line and environment in Abattoir A and in Abattoir B. For Abattoir A, results show that *cpe*-positive carcasses were recovered from 6 out of the 8 visits conducted. Depending on the sampling date, the number of positive carcass samples was as low as 2, and as high as 10.

**Table 3.** Sequential order of the detection of *cpe*-positive broiler chicken carcasses and CSL samples among sampling dates (in 2017) and abattoirs A and B processing line critical steps and environment (CSLs).

Sampling date	Flocks sampled						Environmental sampling (CSLs)	
	1	2	3	4	5		Post-sanitation	Operation
Abattoir A								
February 2^nd^	−	−	−	−	−		−	−
February 9^th^	−	−	−	W	A		−	−
March 29^th^	B	−	B	B, BC	−		−	−
April 20^th^	BE, W, A	W	−	W, A	A		E4	E2, E3
May 11^th^	A	BE, BC, A	BE, BC	−	A		−	E7
May 31^st^	−	−	−	−	−		E7	−
June 27^th^	BE, BC	BE, W	−	−	−		−	E6, E7
June 29^th^	W, A	B, BE, W	BE, W, A	W	A		E3	
Abattoir B								
March 23^rd^	−	−	−	−	−		−	−
March 24^th^	−	−	−	−	−		−	−
April 6^th^	−	−	−	−	−		−	−
April 27^th^	BE, W	−	−	−	−		−	−
May 18^th^	−	−	−	−	−		−	−
June 8^th^	−	W, A	−	−	−		−	−
July 4^th^	−	−	−	−	−		E7	−
July 6^th^	−	−	−	−	−		E6	−

B: bleeding; BE: before evisceration; BC: before chilling; W: water chilling; A: air chilling; CSL: critical sampling location; E1: feather-plucking rubber fingers; E2: conveyor belt between the live receiving and evisceration departments; E3: evisceration machine; E4: floor surface in the evisceration department; E5: conveyor belt before chilling; E6: conveyor belt after chilling; E7: stainless steel equipment surface located in the cut-up room.

### 3.3. Detection of cpe-positive flocks among surveyed abattoirs

At least one positive carcass was found in 21 (27.0%) of the 79 flocks. Higher odds of positivity (odds ratio = 20.3, p < 0.001) were found in flocks slaughtered in abattoir A (50.0% of positive flocks) compared to abattoir B (5.1% of positive flocks). The proportion of positive flocks according to the order of flock slaughtering (from the 1^st^ to the 5^th^ flock slaughtered on a specific sampling date) was 37.5%, 31.3%, 18.8%, 25.0% and 26.7%, respectively.

### 3.4. Detection of cpe-positive samples among CSLs in the slaughter plant environment

Among the 217 collected environmental samples, a total of 11 *cpe*-positive CSLs were identified through the samplings conducted prior to the beginning (prevalence of 4.8%) and at the end of the slaughter operations (prevalence of 5.4%) in both slaughter plants during the project, representing a prevalence of environmental *cpe*-positive samples of 5%. However, *cpe*-positive environmental samples varied among surveyed plants for the samplings conducted prior to the beginning and at the end of the slaughter operations, with a prevalence of 6.1% and 8.9% of positive samples in plant A and 3.6% and 1.8% of positive samples in plant B, respectively. Among the seven CSLs screened, no *cpe*-positive sample was recovered from the samplings conducted on the feather-plucking rubber fingers (E1) and on the conveyor belt before chilling (E5) surfaces. In plant A, the evisceration machine was identified as *cpe*-positive during both PS and OP sampling time points. It should, however, be noted that those positive samples were recovered within an interval of time of two months. Only one *cpe*-positive sample was recovered from the floor surface in the evisceration department (E4) and this sample was collected in plant A. For each sampling visit conducted in processing plant A, the number of *cpe*-positive CSL was variable, with no positive surface identified during 3 visits out of 8, or with a total of 3 positive CSL identified during one of those visits. As for findings resulting from the carcass sampling, most of the environmental samples found positive for *cpe* were identified from the cutting and packaging department. Indeed, 13.3% and 9.4% of the samples collected in this department were found *cpe*-positive in plant A and B, respectively. From those positive environmental samples, a stainless steel surface of an equipment with which the final meat product comes into contact was linked to 3 and to 1 *cpe*-positive samples recovered from the cutting and packaging department of plant A and B, respectively. According to the multivariable exact logistic regression model, no association was found between the *cpe*-positivity of the collected environmental samples and the abattoirs (p = 0.12), the type of surfaces (CSLs) (p = 0.18), or the sampling periods (i.e. post-sanitation v. after the operations, p = 1.00).

### 3.5. Amplified PCR products sequencing, alignment with C. perfringens SM 101 food poisoning reference strain

**Figure 1.**
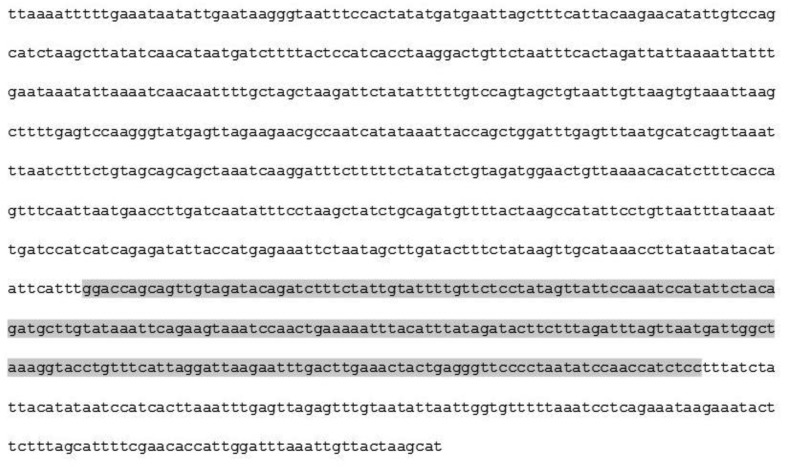
Nucleotide sequence amplified by *cpe*F and *cpe*R primers [5] in *C. perfringens* SM101, locus_tag CPR_0381, *cpe* gene (https://www.ncbi.nlm.nih.gov/nuccore/CP000312.1). Grey box corresponds to the amplified *cpe* gene fragment in the current study.

Two independent PCR products, each compatible with 233 bp size on the agarose gels and originating from two suspected *cpe*-positive broiler chicken carcasses, were purified and sequenced according to the protocol described previously in the Materials and methods section. Sequencing confirmed that both products were 233 bp long. Their alignment with the *cpe* gene sequence of the reference *C. perfringens* strain SM 101 corresponding to the region targeted by both *cpe*F and *cpe*R primers (see Figure 1) showed complete sequence similarity (100%) with SM 101 *cpe* gene, confirming the nature of the amplified products and the presence of *cpe*-carrying *C. perfringens* strains in the analyzed samples of the current study. The amplified 233 bp sequences obtained from the enriched samples were also compared to the publicly available information (accession number CP000312.1). Sequence alignment results showed that the 233 bp sequences were 99% and 100% similar to a 230 and 233 bp long sequence of the enterotoxin gene (*cpe*) found in the chromosome or on plasmids of various reference *C. perfringens* strains (NCBI).

## 4. Conclusions

Studies conducted on *C. perfringens* prevalence in raw poultry meat products at retail in the United States, in Japan, in India and in Canada revealed that between 6% and 97% of the analyzed meat samples were positive for the presence of *C. perfringens*. However, the presence of *cpe* or *cpe*-carrying *C. perfringens* strains among those meat samples was varying between 0% and 15.5% [7]–[10],[12],[36],[37]. To our knowledge, no cross-sectional study has yet been done to investigate the prevalence of *cpe*-positive broiler chicken flocks and carcasses at the slaughter plant level, nor the critical steps of the slaughter process involved in this contamination have been identified [23],[38]. When investigating the intestinal tract of 59 broiler chickens in Switzerland, Tschirdewahn et al. [39] found that 10% of the analyzed intestinal samples were positive for the presence of *cpe*-positive *C. perfringens*. No precision was however given on the number of different flocks that were investigated. As for other foodborne pathogens like *Salmonella* spp. and *Campylobacter* spp., the *C. perfringens* status of the incoming birds most probably plays a role in either directly acting as a source of contamination for their related processed meat products, or could contribute in disseminating this contamination throughout the environment of the processing plant in which, subsequently processed flocks can be cross-contaminated [23]. However, considering that both the numbers of collected and *cpe*-positive samples were relatively low among sampled abattoirs, among sampled CSLs and for both sampling periods, great care is needed to not over-interpret findings pertaining to the role of the abattoir environment in the current study. When investigating the occurrence of generic *C. perfringens* in the scalder, prechill and chill tanks during the slaughtering of three different commercial broiler chicken flocks in one abattoir in Georgia, United States, Craven et al. [40] observed highly variable *C. perfringens* contamination levels between sampled flocks, with one flock accounting for 93% of the *C.* *perfringens*-positive carcasses, with the second flock from which only one positive carcass was identified, and with a third flock from which no *C. perfringens* positive carcass was found. This highly variable contamination level is also one striking observation made for *cpe*-positive *C. perfringens* during the current study. Few upstream steps such as the hatchery, the rearing facility and the transport containers have all been previously recognized for their contribution in the spread of generic *C. perfringens* throughout the poultry production chain [14],[23],[39],[41]. It could then be speculated that the enterotoxigenic counterpart of the *C. perfringens* population could behave similarly, and that the presence of *cpe*-carrying *C. perfringens* at those earlier steps could represent a risk factor for poultry meat contamination at the processing level.

When formulating hypotheses to explain the highly variable generic *C. perfringens* positivity observed in various poultry meat samples analyzed, Guran et al. [5] highlighted the technological differences between slaughterhouses as a contributing factor, with the inevitable cross-contamination occurring during the poultry slaughter process. Sanitation and hygiene conditions were then recognized as a major risk factor. Indeed, it was later on described by other authors that *C. perfringens* forms biofilms that can foster the persistence of this microorganism in the food processing environment, both because of the biofilm structure itself, but also because of the protecting effect it confers to the engulfed cells towards the biocide action of the various disinfectants used in such environments [42]. Humans can also serve as a significant reservoir of a variety of *cpe*-carrying *C. perfringens* genotypes, emphasizing the importance of implementing a good hygiene program in poultry slaughterhouses [43],[44]. As is the case for many large commercial slaughterhouses, there are differences regarding the operation and sanitation program characteristics between the two surveyed slaughter plants in the current study (see Table 1). For instance, quaternary ammonium compounds (QACs) have little sporicidal effect on *C. perfringens* spores, and some previously published research work also suggest that QACs may encourage *Clostridium* vegetative cells to sporulate [45]. The use of such chemicals for carcass decontamination after chilling in abattoir A, and especially the role of the specific equipment required for product application in which biofilms have the potential to form, could be one of the reasons explaining the identification of more *cpe*-positive carcasses after water and air chilling in this slaughter plant [46]. We could also speculate that this residual contamination on chilled carcasses could contribute to the environmental contamination observed in this same abattoir, and that the organic/inorganic acids and hydrogen peroxide compounds used for environmental sanitation are not fully effective at destroying those residual spores or biofilm-encased spore and vegetative forms of the bacterium [42],[46],[47]. From the low environmental *cpe* contamination observed in abattoir B, we could also presume that this environmental contamination could largely result from the presence of vegetative cells originating from the digestive tract of slaughtered birds, this living form of the bacterium being sensitive to the action of QACs during plant sanitation, which is not the case with the spore form of this bacterial species [42],[47]. All of those abovementioned contributing factors should however deserve further investigation.

As opposed to the observations made in the current study, the prevalence of generic *C. perfringens* reported in a previous study showed a decrease along the processing line, with prevalence decreasing from 80% to 20% from the scalder to the chiller tanks [23]. This would be explained by the fact that *C. perfringens* vegetative cells in the poultry slaughterhouse environment are likely to be exposed to environmental stressors such as cold or heat, drying, and sanitizers, most of the time leading to the death of the microorganism [23]. Though this is true for the vegetative counterpart of the *C. perfringens* species, the notable resistance of some enterotoxigenic *C. perfringens* to the control measures used by the food industry (heat, cold, osmotic and nitrite-induced stresses), especially those carrying the *cpe* gene in their chromosome, brings certain nuance to this statement [19],[20],[48]. The heat treatment applied by Craven et al. [23] to the samples recovered at the processing plant further supports this affirmation, as the incidence and numbers of *C. perfringens* heat-resistant spores, were slightly lower than the total counts, indicating a significant contribution of the spore resistance form of this species in the contamination of both the carcasses and the processing plant environment. As the current study was exploratory and aimed solely at better describing the presence of *cpe*-positive *C. perfringens* along the processing line, the combined analysis of both the non-heat-treated and heat-treated tubes for the samples recovered unfortunately precluded us from further describing the relative importance of each of the vegetative and spore forms of CPE-producing *C. perfringens* in their capacity to contaminate broiler chicken carcasses. This should be addressed in future research work. The more important bacterial load at initial steps of the processing line might also have created a less favorable environment for the recovery of *cpe*-bearing *C. perfringens*, as the microorganism was rather competitively grown according to the amount of nutrients available in the enrichment media (FTG medium) [49]. Conversely, a reduction in the number of competitive microorganisms, combined with the survival of spores along the process, as well as in the processing environment could explain why more *cpe*-positive carcasses were recovered after both immersion-water (W) and dry-air (A) chilling steps in the current study [23].

The low prevalence of *cpe*-positive samples recovered from both the sampled broiler chicken carcasses and the slaughter plant environment are unfortunately a limiting factor in the ability to establish a link between the processed carcasses and the slaughter plant environment. However, based on the results presented in Table 3 and pertaining to abattoir A, it is worth reporting that visits for which no CSL was identified as *cpe* positive during both PS and OP samplings showed the lowest numbers of *cpe*-positive broiler chicken carcasses. Based on those results, the *cpe* status of a broiler chicken flock, prior to slaughter, and the fact that those birds would act as a source of contamination by fostering the accumulation of *cpe*-carrying *C. perfringens* strains along the processing line, from the live receiving department to the packaging room, is worthy of particular attention. The isolation of *cpe*-positive *C. perfringens* strains from the positive samples recovered will help establish this link. Further investigations are also required in order to better define the factors affecting this incidence among slaughter plants, as well as between broiler chicken flocks slaughtered in a same processing establishment. In addition, as only two slaughter plants among the 57 federally-inspected poultry processing establishments were sampled in the current study, documenting the presence of enterotoxigenic *C. perfringens* at the Canadian level would help better define the burden of *C. perfringens* food-borne disease outbreak attributable to poultry in Canada. Thus, the role of the incoming birds, of the abattoir's slaughter and hygiene practices and of the *cpe*-carrying *C. perfringens* strains presumably persisting in the processing plant environment need to be better defined in order to implement the appropriate prevention and control strategies.
